# Bibliometric analysis on global Catha edulis (khat) research production during the period of 1952–2014

**DOI:** 10.1186/s12992-015-0124-x

**Published:** 2015-09-04

**Authors:** Sa’ed H. Zyoud

**Affiliations:** Poison Control and Drug Information Center (PCDIC), College of Medicine and Health Sciences, An-Najah National University, Nablus, 44839 Palestine; Department of Clinical and Community Pharmacy, College of Medicine and Health Sciences, An-Najah National University, Nablus, 44839 Palestine

**Keywords:** Bibliometric analysis, Catha edulis, *h*-index, Khat, *Scopus* database

## Abstract

**Background:**

Publication of scientific articles related to khat (*Catha edulis*) in peer-reviewed journals is considered a measure of research productivity. The principal objectives of this study were to quantify the research contribution related to khat at the global level, as well as to determine its relative growth rate, collaborative measures taken, productivity at the institutional level, and the most prolific journals publishing on the topic.

**Methods:**

On the basis of title words related to khat, publications were identified for all data in Scopus bibliographic database’s history up to December 31, 2014. The research productivity for the top 10 countries was evaluated in relation to the population size and gross domestic product (GDP) in 2013.

**Results:**

The criteria were met by 651documents published in 51 countries. The largest number of articles associated with khat was from the UK (15.2 %), followed by Yemen (10.3 %), the USA (9.7 %) and Ethiopia (9.1 %). Ethiopia, Yemen, and Kenya had the highest productivity of publications after standardization by population size and GDP. Furthermore, Yemen achieved the highest number of collaborations, by having researchers from 19 countries. Ethiopia followed, having researchers from 16 countries.

**Conclusions:**

This bibliometric study provides a demonstration for the worldwide research activity regarding khat. The number of articles related to khat has increased rapidly over the last 10 years. The present study is a good starting point to evaluate research activity in the field of khat. Although the data shows a promising increase in the research activity, the quantity of khat-related research is still too little compared to the massive use of khat in certain countries.

## Background

Khat (*Catha edulis*) is a stimulant plant grown commonly in Southern Arabia and East Africa. The leaves of the khat shrub are characterized by an aromatic odour, with an astringent and slightly sweet taste [1]. The leaves and buds of khat are chewed to reach a state of euphoria and excitement [2].

The main active ingredients of khat are cathine and cathinone, thus khat chewing may have various different compounds with different effects [3]. Chewing khat mainly affects the user’s gastro-intestinal system and the central nervous system (CNS). Tolerance to and dependence on khat and its psychiatric symptoms may be observed as effects on the CNS [2–4, 1, 5, 6]. The World Health Organization (WHO) categorized khat as a drug of abuse that can lead to mild to moderate psychological dependence but to a lesser degree than nicotine and alcohol [7], and the WHO does not deem khat to be as dangerously addictive as cocaine [8]. The khat chewing habit has spread with African and Arabian immigrants to Australia, Europe, and Asia, as well as to the United States, and it is becoming a global phenomenon [9]. It is legal for sale and production in some countries but is a controlled or illegal substance in others. The use of khat is accepted within Yemeni, Ethiopian, Eritrean, Djiboutian, Kenyan, Somali and Ugandan cultures [3, 6] but is prohibited in the USA, France, Sweden, and Switzerland. Khat use had been tolerated in the Netherlands and in the UK for a long time, but in 2012, the recreational use of khat was prohibited in the Netherlands, and the UK followed the prohibition in 2014 [10].

Although quite a few bibliometric studies have been carried out on the field of substance abuse [11–17], they have failed to reveal any data concerning the evaluation of scientific research output regarding khat at the global or regional level. The evaluation of scientific research at global, regional, or national levels is necessary to improve its research productivity [18]. The bibliometric approach is a research method used to evaluate the current state of scientific research in certain areas by conducting a precise analysis to obtain statistics that can be considered indicators of achievement that allow researchers to recognise and improve their research [19, 20]. This type of analysis utilizes quantitative methods and statistics to describe publications within a given field, journal, institution, or country [21, 22, 20]. The main objectives of this bibliometric study were to examine the publication pattern of khat at the global level, as well as to determine the publications’ relative growth rate, collaborative measures, productivity at the institutional level, and the most prolific journals publishing on khat, as retrieved from the *Scopus* database. It is hoped that the results of this study will contribute to quality improvement in future research on khat. This study can be used as a baseline data point to direct national and international policies regarding khat. For example, khat needs to be introduced as one potential substance of abuse in many teaching materials and rehabilitation centres.

## Methods

### Search strategy

Scientific output was assessed based on a methodology designed and used in previous bibliometric studies [23, 20, 24–30]. SciVerse *Scopus* was used to collect data pertaining to the current study because it includes all MEDLINE journals, and it contains all authors’ country affiliations, which were needed for seeing international collaborations, institutional phenomena, and countries’ production rates. Furthermore, Scopus is considered to be the largest international multidisciplinary database in the world, and it covers a wider range of journals from developed and developing countries than does MEDLINE or Web of Science [31, 32].

The terminology used for *Catha edulis* varies between regions and includes names such as qat in Yemen and in the Kingdom of Saudi Arabia (KSA), mirra in Kenya, khat in Ethiopia, and jaad or qaad in Somalia. However, in most of the literature, it is known as khat [33]. The keywords entered into the search engine were retrieved from previous research related to khat [1, 33–35]. All of the following selected keywords were entered in the field for article titles: “khat”, or “jaad”, or “mirra” or “qaad”, or “mairungi” or “qat” or “Catha edulis” or “catha”. The scope of the research went from as far back as Scopus has archived records (1952) through to December 31, 2014. Documents that were published as errata were excluded. In addition, those documents in which the concepts were not related to khat were excluded. Articles from 2015 were excluded because Scopus has not yet archived all of these issues. The collected data were used to create the following measurements: growth rate, collaborative measures, productivity at the institutional level, the most productive authors, the most prolific countries with citation patterns, and the most prolific journals [23, 20, 24, 25]. All of these measurements were ranked according to the order that is now popularly called standard competition ranking (SCR), as in previous similar bibliometric studies [20, 24, 25]. The quality of publications related to khat was measured using the *h*-index, which was established by Jorge Hirsch in 2005, where index h is defined as the number of papers with a citation number more than or equal to h [36]. Furthermore, the quality of the journals was assessed by two indicators: the impact factor (IF) using the Journal Citation Report (JCR; Web of Knowledge) 2013 and the *SCImago Journal Rank* (SJR). Additionally, publication activity was adjusted for the top 10 countries by using the adjustment index (AI) formula [37, 24, 28]. The research productivity for the top 10 countries was evaluated in relation to the population size and the gross domestic product (GDP) in 2013 [38].

### Statistical analysis

Data were entered in a Microsoft Excel sheet and then transferred to the Statistical Package for Social Science programs (SPSS, V.15) for data management and analyses. Data are presented as medians (with interquartile ranges) or as numbers with percentages.

## Results

**Fig. 1:**
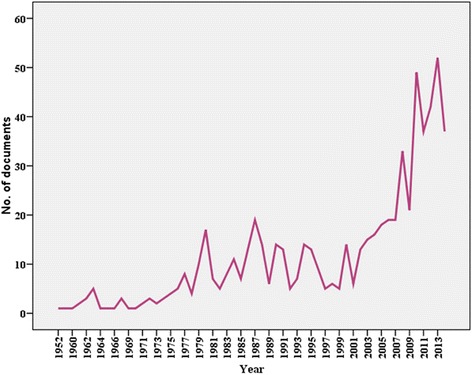
Total articles included in a bibliometric analysis of worldwide publications related to khat from 1952 to 2014

There were 651 articles meeting the search criteria from 1952 to 2014. We identified 491 (75.4 %) articles of original research, 45 (6.9 %) letters to the editor, 39 (6.0 %) reviews, and 76 (11.7 %) articles that were categorized as other types of publications, such as notes or editorials. The numbers of articles related to khat soared rapidly during the last decade. Before 2002, the number of annual publications related to khat was less than 280 papers, which has grown much more rapidly since 2008 (Fig. 1). The first document related to khat was published by Baird in *East African Medical Journal* in 1952 [39]. The great majority of articles retrieved were in English (90.2 %). Other relatively frequent languages were French (3.8 %) and German (2.9 %).

All of the extracted publications were published in 51 countries. The largest number of articles associated with khat was from the UK (15.2 %), followed by Yemen (10.3 %), the USA (9.7 %), and Ethiopia (9.1 %); (Table 1). Ethiopia, Yemen, and Kenya had the highest productivity of publication after standardization by population size and GDP (Table 1). The total number of citations was 7976, giving an average citation per item of 12.3. The median (interquartile range) was 5 (1–16). The highest median (interquartile range) number of citations was 16 (5–28) for Italy, followed by 10 (2–33) for Switzerland. The *h-*index of the retrieved documents was 44 (44 documents had been cited at least 44times for the period of study). The highest *h-*index was 22 for the UK, followed by 17 for Yemen and for Switzerland. Furthermore, Yemen achieved the highest number of collaborations, with collaborating researchers from 19 countries. Ethiopia followed, having collaboration among researchers from 16 countries. Yemen had the highest percentage (67.2 %) of documents in collaboration with international authors, followed by 60 % for Egypt, and 53.1 % for Germany (Table 1).

**Table 1:** The top 10 ranking of the most productive countries in publishing the largest number of articles associated with khat during the period from 1952 to 2014

SCR	Countries	No. of articles (%)	*h-*index	Median (Q1-Q3) of citation	Average of citation	Collaborations with foreign countries	Number (%)^a^ of documents with international authors	Adjustment index ^b^
1st	UK	99 (15.2)	22	7 (2–20)	15.2	13	27 (27.3)	2.4
2nd	Yemen	67 (10.3)	17	4 (0.0-21)	12.9	19	45 (67.2)	45.5
3rd	USA	63 (9.7)	15	5 (1–4)	10.7	10	25 (39.7)	1.2
4th	Ethiopia	59 (9.1)	12	1 (0.0-11)	7.9	16	20 (33.9)	116.8
5th	KSA	55 (8.4)	11	2 (1–9)	6.4	11	16 (29.1)	2.1
6th	Switzerland	47 (7.2)	17	10 (2–33)	22.6	1	4 (8.5)	0.6
7th	Germany	32 (4.9)	12	9 (1–24)	15.8	15	17 (53.1)	0.7
8th	Kenya	23 (3.5)	10	8 (4–15)	11.3	3	6 (26.1)	18.5
9th	Egypt	20 (3.1)	5	1 (0–5.8)	3.1	7	12 (60.0)	6
10th	Italy	19 (2.9)	11	16 (5–28)	18.3	6	4 (21.1)	0.5

*KSA* The Kingdom of Saudi Arabia, *SCR* Standard Competition Ranking, *USA* The United States of America, *UK* The United Kingdom, *Q1–Q3* lower quartile - upper quartile

^a^Percentage of documents with international authors from the total number of documents for each country

^b^An adjustment index (AI) was measured using the following formula: AI = [total number of publications for the country / GDP per capita of the country]*1,000, where GDP per capita = GDP/population of the country

Table 2 shows the data for the most prolific journals in the field of khat. Forty-eight documents (7.4 %) were published in *Journal of Ethnopharmacology,* whereas 14documents (2.2 %) were published in *East African Medical Journal*, followed by9 documents (1.4 %) for each of *Saudi Medical Journal* and *Forensic Science International.* Four journals from the most prolific journals in the field of khat had no official IF and only four journals had SJR >1 (Table 2).

**Table 2:** Ranking of the top 10 journals in which articles associated with khat were published worldwide

SCR^a^	Journal	Frequency (%)	IF (2013)^b^	SJR
1st	Journal of Ethnopharmacology	48 (7.4)	2.939	1.149
2nd	East African Medical Journal	14 (2.2)	NA	0.152
3rd	Saudi Medical Journal	9 (1.4)	0.554	0.269
3th	Forensic Science International	9 (1.4)	2.115	1.293
5th	Bulletin on Narcotics	8 (1.2)	NA	NA
6th	Lancet	7 (1.1)	39.207	11.563
6th	Journal of the Chemical Society Perkin Transactions 1	7 (1.1)	NA	NA
6th	BMC Public Health	7 (1.1)	2.321	1.233
6th	Planta Medica	7 (1.1)	2.339	0.791
6th	Medecine Tropicale	7 (1.1)	NA	0.174

*SCR* standard competition ranking; *SJR SCImago* Journal Rank, *NA* not available, *IF* impact factor

^a^Equal journals have the same ranking number, which leaves a gap in the ranking numbers

^b^The impact factor was reported according to the Institute for Scientific Information (ISI) journal citation reports (JCR) 2013

Table 3 shows the scientific articles’ areas of interest. Medicine was the most researched topic, represented by 366 (56.2 %) articles. The second most researched topic was pharmacology, toxicology and pharmaceutics, represented by 179 (27.5 %), followed by biochemistry, genetics, and molecular biology with 88 (13.5 %) articles.

**Table 3:** Ranking of the top 10 interest areas of the published articles associated with khat

SCR^a^	Areas of interest	N (%)^b^
1st	Medicine	366)56.2)
2nd	Pharmacology, Toxicology and Pharmaceutics	179 (27.5)
3rd	Biochemistry, Genetics and Molecular Biology	88 (13.5)
4th	Social Sciences	56 (8.6)
5th	Chemistry	53 (8.1)
6th	Agricultural and Biological Sciences	43 (6.6)
7th	Neuroscience	42 (6.5)
8th	Environmental Science	26 (4.0)
8th	Psychology	26 (4.0)
10th	Dentistry	23 (3.5)

*SCR* standard competition ranking

^a^Equal areas of interest have the same ranking number, which leaves a gap in the ranking numbers

^b^Total exceeds 100 % as data are overlapping due to multidiscipline interactions

A list of the most cited articles in the field of khat is shown in Table 4 [40–47, 1, 48]. Table 5 presents the 10 institutions producing the most khat research articles. The most productive institution was Sana’a University, Yemen (8.0 % of total publications); followed by Addis Ababa University, Ethiopia (4.5 %), and King Saud University College of Pharmacy, KSA (4.0 %). Table 6 provides the names of the most prolific authors who have contributed at least ten articles in the field of khat.

**Table 4:** Top 10 ranking of cited articles in *Scopus* related to khat worldwide

SCR^a^	Authors with year of publication	Title	Source title	Cited by	Article type
1st	Halbach 1972 [40]	Medical aspects of the chewing of khat leaves	*Bulletin of the World Health Organization*	145	Review
2nd	Kalix and Braenden 1985 [41]	Pharmacological aspects of the chewing of khat leaves	*Pharmacological Reviews*	142	Review
3rd	Al-Motarreb et al. 2002 [42]	Khat: Pharmacological and medical aspects and its social use in Yemen	*Phytotherapy Research*	128	Review
4th	Brenneisen et al. 1990 [43]	Amphetamine-like effects in humans of the khat alkaloid cathinone	*British Journal of Clinical Pharmacology*	116	Original article
5th	Widler et al. 1994 [44]	Pharmacodynamics and pharmacokinetics of khat: A controlled study	*Clinical Pharmacology and Therapeutics*	110	Original article
6th	Toennes et al. 2003 [45]	Pharmacokinetics of cathinone, cathine and norephedrine after the chewing of khat leaves	*British Journal of Clinical Pharmacology*	104	Original article
7th	Kalix 1990 [46]	Pharmacological properties of the stimulant khat	*Pharmacology and Therapeutics*	90	Review
8th	Luqman and Danowski 1976 [47]	The use of khat (Catha edulis) in Yemen. Social and medical observations	*Annals of Internal Medicine*	84	Review
9th	Cox and Rampes 2003 [1]	Adverse effects of khat: A review	*Advances in Psychiatric Treatment*	82	Review
10th	Odenwald et al. 2005 [48]	Khat use as risk factor for psychotic disorders: A cross-sectional and case–control study in Somalia	*BMC Medicine*	77	Original article

*SCR* standard competition ranking

^a^Equally cited articles have the same ranking number, which leaves a gap in the ranking numbers

**Table 5:** Ranking top 10 highly productive institutions that most frequently published articles associated khat worldwide

SCR^a^	Institution, country	No. of documents (%)
1st	*Sana’a University, Yemen*	52 (8.0)
2nd	*Addis Ababa University, Ethiopia*	29 (4.5)
3rd	*King Saud University, KSA*	26 (4.0)
4th	*University of Nairobi, Kenya*	20 (3.1)
4th	*Universite de Geneve, Switzerland*	20 (3.1)
6th	*University of Minnesota, USA*	19 (2.9)
7th	*Jazan University, KSA*	15 (2.3)
8th	*University of Nottingham, UK*	14 (2.2)
9th	*Jimma University, Ethiopia*	12 (1.8)
10th	*University of Kent, UK*	10 (1.5)
10th	*Universitat Bern, Switzerland*	10 (1.5)

*SCR* standard competition ranking, *KSA* The Kingdom of Saudi Arabia, *UK* The United Kingdom

^a^Equal institutions have the same ranking number, which leaves a gap in the ranking numbers

**Table 6:** The 10 most-productive authors

SCR^a^	Author name	Total number of articles (%)	Affiliation
1st	Kalix, P.	32 (4.9)	Universite de Geneve Faculte de Medecine, Department of Pharmacology, Geneve, Switzerland
2nd	Al’Absi, M.	17 (2.6)	University of Minnesota Twin Cities, Department of Family & Community Medicine, Minneapolis, USA
3rd	Al-Meshal, I.A.	16 (2.5)	King Saud University College of Pharmacy, Department of Pharmacognosy, Riyadh, KSA
4th	Al-Habori, M.	14 (2.2)	Sana’a University, Department of Biochemistry and Molecular Biology, Sana’a, Yemen
5th	Brenneisen, R.	13 (2.0)	Universitat Bern, Department of Clinical Research (DCR), Bern, Switzerland
5th	Murray-Lyon, I.M.	13 (2.0)	Department of Gastroenterology, Chelsea and Westminster Hospital, 369 Fulham Road, London SW10 9NH, UK
5th	Crombie, L.	13 (2.0)	University of Nottingham, Department of Chemistry, Nottingham, UK
5th	Tariq, M.	13 (2.0)	Department of Pharmacology, College of Pharmacy, King Saud University, P.O. Box 2457, Riyadh-11451, KSA
9th	Whiting, D.A.	11 (1.7)	University of Nottingham, Department of Chemistry, Nottingham, UK
10th	Nakajima, M.	10 (1.5)	University of Minnesota Medical School, 1035 University Drive, Duluth, Minnesota 55812, USA
10th	Ageel, A.M.	10 (1.5)	King Saud University, College of Pharmacy, Department of Pharmacology, Riyadh, KSA

*SCR* standard competition ranking, *KSA* The Kingdom of Saudi Arabia, *USA* The United States of America, *UK* The United Kingdom

^a^ Equal authors have the same ranking number, which leaves a gap in the ranking numbers

## Discussion

In this study, I obtained some significant points regarding the research productivity throughout the period between 1952 and 2014. The number of articles related to khat has increased rapidly over the last 10 years. As far as I am aware, this is the first study carried out to evaluate the quantity and quality of khat-based research from all countries in the world. The number of published articles was used as an indicator of the quantity of research activity in the field of khat. The number of citations in an h-index and the impact factors were used as quality indicators [49–51]. This bibliometric study focused primarily on assessing the publication pattern of articles about khat at the global level, the productivity of particular institutions, collaborative measures, and the utility of various journals to the field of khat, which is known as a sub-area in the field of substance abuse. Understanding of how research related to khat has progressed is important in reducing morbidity and mortality related to substance abuse. Such understanding of research activity in the field of khat is helpful in developing an effective policy to respond to this progress, as well as it gives an opportunity for policymakers to gain public and political support for their measures [6].

Ina critical review conducted in 2007, Warfa et al. identified a large amount of evidence on khat, most of which related to khat and to mental health, its adverse effects, and its harms to users and to society [52]. The current bibliometric study also adds to the bibliometric literature in the field of substance abuse [11–15, 17, 16].

It is interesting to note that research performance in the field of khat has been neglected in some countries. However, researcher should grow because khat use has an increasing global market and a documented economic value similar to other harvests, such as coffee, cacao, and tea. During the last few decades, khat use has an increased global importance due to migration, leading to an increase in health problems among its users. The khat trade has a complex delivery system, and thus efforts to prohibit it would need information about the probable risks of a black market developing if khat becomes criminalized [53].

The most obvious finding to emerge from the analysis is that research collaborations in the field of khat, compared to other fields, has been neglected or low in most countries [54, 23–25]. Contributions in research output from collaboration of different world regions with Yemen and Ethiopia were evident. Several studies have found that there is a positive correlation between international collaboration and research output [55–57]. The benefits of increasing collaborations are that it leads to easier access to financing, more opportunities to achieve a higher research productivity [58–60], and it facilitates translation research expertise to countries that require such [28, 24, 61]. As the world faces the truth of khat use as a phenomenon, efforts to put it under global principles and achievable control at national levels should increase. However, these attempts will involve comprehensively reflecting on all the aspects that the expansion of khat consumption and production highlight as a global phenomenon [53]. We need more collaboration in research in order to recognize the actual size and the reasons related to the rapid increase of khat use in some countries such as Yemen and Ethiopia. Continuous research for evaluating the outcomes is another important aspect of knowing more regarding the community status before and after the application of health education programs, public awareness, and information campaigns.

The results of this study show that publications related to khat were 12.3 citations per article. This result is in line with those of previous studies, especially on those that were published in toxicological journals [62–64, 29, 28], and within the average citation range of integrative and complementary medicine journals [65–70]. Several recent studies using the same bibliometric technique indicated that the average citation rate for publications on electronic cigarettes was 6.4 citations per article [20]; on calcium channel blockers, 9.1 [27]; on paracetamol,12.3 [28]; and on narghile tobacco smoking, 13 [23].

The 10 most prolific countries that published articles on khat include many new countries that are usually not as recognizable to readers as other scientific research productivity rankings [71]. As shown in this study, the behaviour of each country, in terms of scientific research output, differs. In countries such as the UK, the total number of scientific publications related to khat output accounted for more than 15.2 % of the global research output. Therefore, this research activity might be dependent on the population size, the GDP, and the level of research activity [72, 28]. In this study, after adjusting for economy and population power, the ranking of countries’ research productivity differed clearly from those based on absolute research production. After adjusting for the national GDP per capita and population, Ethiopia, Yemen, and Kenya had the highest research productivity. No similar study has been found in the literature to this point, thus I was unable to interpret this finding in light of other results. Based on these results, countries with rapidly growing economies and with large population sizes were found to be among the main factors related to research productivity. These findings suggest that rapid economic growth in these countries may result in more research investments and funding support, and may lead to enhance research activity related to khat. The sale and consumption of khat are legal in some countries, including Ethiopia, Kenya and Yemen, which fact might contribute to scientific research productivity.

A bibliometric study was conducted in 10 European countries to examine publications in the field of addiction in comparing to the same type of published data from the USA during the years 2001–2011 [11]. It was found that the absolute increase in publications in the field of addiction was higher in Europe as a whole (an increase on average of 113.8 papers per year) than in the USA. A bibliometric study was also conducted by a number of scholars to examine the worldwide scientific research output in the field of alcohol drinking and alcohol-related problems during the years 2001–2011 [16]. The researchers found that research about alcohol is an integrated field, with an average of 4820 documents published each year in *Scopus* and in MEDLINE. The bibliometric analysis in this study showed that scientific research output in the field of khat is lagging behind in substance abuse research and the sharing to global research in substance abuse is low.

The present study has several limitations that need to be stated, most of which were mentioned in previous similar bibliometric studies [70, 37, 29, 30]. Studies on khat that were indexed in databases other than *Scopus* may not have been included. Gaillard demonstrated that some African researchers have published their work in local journals that are not indexed in the international citation databases [73]. Another limitation is that some articles did not mention khat or related expressions in their titles, so it is possible that not all articles about khat were considered.

### Conclusions and research-policy considerations

In conclusion, this bibliometric study provides a demonstration of worldwide research activity on khat. The number of articles related to khat has increased rapidly for the last 10 years. The present study is a good starting point to evaluate research activity in the field of khat. Although the data shows a promising increase in the research activity, the quantity of khat-related research is still too little compared to the massive use of khat in certain countries. The quantity and quality of khat research can be enhanced by providing more collaboration with international research projects related to khat use.

Governments of countries in which khat is being used, such as Ethiopia, Yemen, and Kenya, need to implement policies regarding the cultivation of khat and restriction on the areas that are used for khat cultivation. Furthermore, the shipping of khat to other countries, even for personal use, must be considered a legal violation and needs to be prohibited at the global level. Governments need to launch awareness programs and campaigns to fight this social phenomenon, which has surpassed tobacco smoking in some countries. Finally, it is recommend that (i) more research efforts be invested in khat regarding its neuronal and oral health impacts; (ii) communities need to implement new regulations to limit khat use among individuals (iii) and finally, countries make efforts to change their citizens’ khat chewing behaviour by implementing community-based primary prevention activities and by improving the community economic status.

## Abbreviations

AI: Adjustment index
CNS: Central nervous system
GDP: Gross domestic product
IFs: Impact factors
ISI: Institute for Scientific Information
JCR: Journal Citation Report
KSA: Kingdom of Saudi Arabia
Q1–Q3: Lower quartile upper quartile
SCR: Standard competition ranking
SJR: *SCImago* Journal Rank
SPSS: Statistical Package for Social Sciences
USA: United States of America
UK: United Kingdom
